# The complete chloroplast genome of *Zelkova serrata* and its phylogenetic position within Ulmaceae

**DOI:** 10.1080/23802359.2020.1768947

**Published:** 2020-05-28

**Authors:** Lingdan Wang, Riqing Zhang, Maolin Geng, Yufeng Qin, Hailong Liu, Mimi Li

**Affiliations:** a Central South University of Forestry and Technology, Changsha, China; b Institute of Botany, Jiangsu Province and Chinese Academy of Sciences, Nanjing, China; c Guangxi Key Laboratory of Superior Timber Trees Resource Cultivation, Guangxi Forestry Research Institute, Nanning, China; d The Jiangsu Provincial Platform for Conservation and Utilization of Agricultural Germplasm, Nanjing, China

**Keywords:** *Zelkova serrata*, *Zelkova*, Ulmaceae, chloroplast genome

## Abstract

The complete chloroplast genome sequence of the Tertiary relict tree *Zelkova serrata* was reported in this study. The chloroplast genome is 158,875 bp in length with a typical angiosperm quantitative structure consisting of a large single copy (87,412 bp) and a small single copy (18,683 bp) separated by a pair of inverted repeat (26,390 bp). Genome annotation revealed a total of 129 genes comprising 84 protein-coding genes, 37 tRNA genes, and eight rRNA genes. Phylogenomic analysis based on the whole plastomes indicated that *Z. serrata* and *Z. schneideriana* formed a well-supported monophyletic clade sister to genus *Ulmus* in Ulmaceae.

*Zelkova serrata* (Thunb.) Makino is an economically and ecologically important Tertiary relict tree distributed endemic to East Asia, including Japan, Korea and China (Fu et al. 2013; Naciri et al. 2019). It is widely used as timber and landscape tree. The leaves and barks are used in traditional herbal medicine for the treatment of various diseases. Its whole tree extraction is also used to treat cancer (Kang and Jang 2012). Here, we reported the plastome sequence of *Z. serrata*, which will provide a valuable genetic resource for further studies, such as genetic diversity, species delimitation, and reconstructing the phylogeny of Ulmaceae.

The leaf materials of *Z. serrata* were collected from Changsha, Hunan province, China (28°8’N, 113°0’E). The specimen was deposited in Central South University of Forestry and Technology (Voucher No.: 201709001). The total genomic DNA was extracted from the silica gel dried leaves using Plant Genomic DNA Kit DP305 (Tiangen, Beijing) and sequenced on Illumina Hiseq Xten platform (San Diego, CA). The raw data were directly assembled using NOVOPlasty 2.7.2 (Dierckxsens et al. 2017) with *Z. schneideriana* chloroplast genome sequence (MK096789) as a reference. Gene annotation was performed by GeSeq (Tillich et al. 2017) and confirmed manually for the start and stop codons in Geneious 11.1.5 (Kearse et al. 2012).

The plastome sequence of *Z. serrata* (Genbank accession number: MT409427) was assembled to 158,875 bp in length. It possessed a typical angiosperm quadripartite and circular structure including a large single copy (LSC, 87,412 bp), a small single copy (SSC, 18,683 bp) and a couple of inverted repeat (IRs, 26,390bp). A total of 129 genes were identified, containing 84 protein-coding genes (CDS), 37 tRNA, and eight rRNA. Seven CDS, seven tRNA and four rRNA were repeated in IR regions. The overall GC content of *Z. serrata* plastome was 35.6% with 42.4%, 33.0%, and 28.4% in the IRs, LSC, and SSC, respectively.

To investigate the phylogenetic relationships within Ulmaceae, additional 13 chloroplast genomes were retrieved from GenBank. Whole plastome sequences alignment was performed using the MAFFT 7.409 (Katoh and Standley 2013). We reconstructed maximum likelihood (ML) phylogeny tree using RAxML 8.2.4 (Stamatakis 2014) with GTR GAMMA model under 1000 bootstrap replicates using *Chaetachme aristata* (MH118120) as an outgroup (Figure 1). The phylogenetic result revealed that *Z. serrata* was sister to *Z. schneiderianais*, and then formed a robust clade with genus *Ulmus* in Ulmaceae.

**Figure 1.**
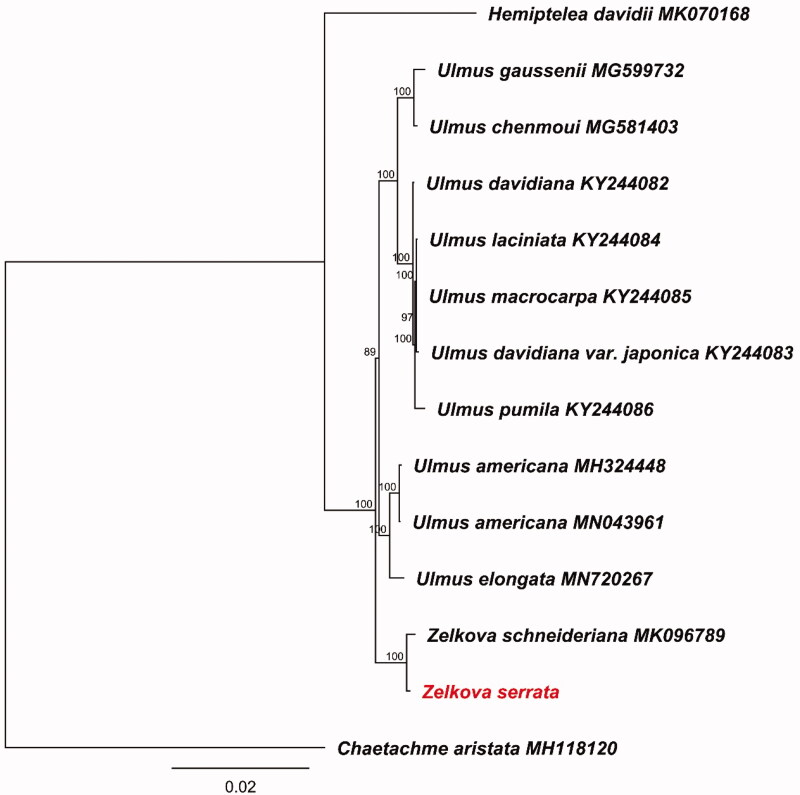
Maximum likelihood tree based on whole chloroplast genomes.

## Data Availability

The data that support the findings of this study are openly available in GenBank (https://www.ncbi.nlm.nih.gov) with the accession number is MT409427.
